# Data imbalance in cardiac health diagnostics using CECG-GAN

**DOI:** 10.1038/s41598-024-65619-8

**Published:** 2024-06-26

**Authors:** Yang Yang, Tianyu Lan, Yang Wang, Fengtian Li, Liyan Liu, Xupeng Huang, Fei Gao, Shuhua Jiang, Zhijun Zhang, Xing Chen

**Affiliations:** 1 School of Electronic Information Engineering, Changchun University of Science and Technology, Changchun, 130022 China; 2 Changchun University of Architecture and Civil Engineering, Changchun, 130607 China; 3 Jilin Province Advanced Control Technology and Intelligent Automation Equipment Research Engineering Lab, Changchun, 130022 China; 4 Tongfang Nuctech Co., Beijing, 100084 China; 5 Department of Cardiology, FAW General Hospital, Changchun, 130011 Jilin China

**Keywords:** Heart disease, Generative adversarial networks, Unbalanced data, Multi-class classification, Electrocardiogram, Computational biology and bioinformatics, Computational models, Computational neuroscience, Data processing, Databases, Machine learning

## Abstract

Heart disease is the world’s leading cause of death. Diagnostic models based on electrocardiograms (ECGs) are often limited by the scarcity of high-quality data and issues of data imbalance. To address these challenges, we propose a conditional generative adversarial network (CECG-GAN). This strategy enables the generation of samples that closely approximate the distribution of ECG data. Additionally, CECG-GAN addresses waveform jitter, slow processing speeds, and dataset imbalance issues through the integration of a transformer architecture. We evaluated this approach using two datasets: MIT-BIH and CSPC2020. The experimental results demonstrate that CECG-GAN achieves outstanding performance metrics. Notably, the percentage root mean square difference (PRD) reached 55.048, indicating a high degree of similarity between generated and actual ECG waveforms. Additionally, the Fréchet distance (FD) was approximately 1.139, the root mean square error (RMSE) registered at 0.232, and the mean absolute error (MAE) was recorded at 0.166.

## Introduction

Heart disease has become the leading cause of death globally, with a notable increase in its prevalence among younger populations in recent years. Furthermore, significant global demographic shifts, such as population aging and growth, have been observed over the past three decades. According to the World Heart Report 2023 published by the World Heart Federation, cardiovascular disease (CVD) fatalities have escalated from approximately 12.1 million in 1990 to about 20.5 million in 2021. Sudden cardiac death and ischemic heart disease constitute 85% of these deaths worldwide. Diagnosis and treatment of such diseases predominantly depend on professional analysis of electrocardiograms (ECGs), which record the heart’s electrophysiological activity over time through skin-placed electrodes. ECGs are increasingly recognized as vital in cardiology therapeutics. However, the medical field faces notable challenges: (1) Human cardiac activity is constantly and rapidly changing, making manual data analysis by medical professionals highly challenging; (2) Machine learning-based detection algorithms necessitate extensive datasets for effective modeling, and manual data labeling incurs substantial time costs and raises patient privacy concerns.

Machine learning algorithms are now gradually making a difference in the field of medical diagnostics with their automatic modelling benefits, such as BP neural networks, decision trees, temporal memory networks and other methods. However, these methods necessitate extensive ECG data for training purposes. The classification and labeling of ECGs involve considerable time and resources from medical professionals. For instance, constructing a cardiac disease classification model requires a substantial dataset of ECG samples. This need contrasts sharply with the prevalent scarcity of medical data, a factor that has impeded progress in related research.

In recent years, the advent of generative adversarial networks (GANs) has significantly expanded dataset diversity across various fields. GANs have found widespread application in image generation for producing high-resolution images and other uses. In the medical domain, Delaney et al.^1^ have both qualitatively and quantitatively shown that GAN architectures can effectively generate diverse time-series signals. Hazra D et al.^2^ introduced SynSigGAN, an innovative GAN model for creating various synthetic biomedical data, demonstrating high correlation coefficients to aid healthcare system development and automation. Zhu F et al.^3^ developed a GAN model based on bi-directional long and short term memory networks and convolutional neural network (BiLSTM-CNN GAN), capable of generating ECG data closely resembling actual ECG recordings. Li X et al.^4^ introduced the transformer-based model called TTS-GAN, utilizing transformer architecture in both generator and discriminator. This model employs visualization and dimensionality reduction techniques to show the similarity between real and generated time series data. Adib E et al.^5^ combined a conditional GAN with WGAN-GP for data augmentation in arrhythmia classification, validating their model with recall, confusion matrix, and accuracy metrics. However, there are still some key issues in these studies:

- (1) The imbalance in current heart rate abnormality datasets significantly hampers the effectiveness of existing classification methods. The current imbalance in heart rate anomaly datasets severely impacts the validity of existing classification methods. This leads to low actual accuracy of scarcity types when assessing heart rate metrics.
- (2) Existing generative ECG model effects still suffer from the problem of imbalance, which exacerbates the negative impact of model performance when training the classification model, resulting in the existing heart rate classification algorithms being heavily biased towards the majority class results, making it difficult to differentiate between new anomalous data.
- (3) Predominantly, existing heart rate generation models utilize recurrent neural networks (RNNs) and convolutional neural networks (CNNs), with a primary focus on sequential output. This approach is both time-intensive and inefficient, leading to cumulative generation errors and resulting in jittery waveforms.

To address these issues, we propose a novel heart rate generation strategy utilizing conditional generative adversarial networks. This model integrates a transformer architecture with conditional constraints, enabling the generative adversarial network to more accurately approximate real data distributions. This approach not only captures a broader range of scarce data distributions but also preserves data diversity. Consequently, it mitigates the performance degradation of classification models caused by data imbalances and addresses the issues related to prolonged output times and subpar results in existing models.

## Methods

### Analyses of imbalanced data distribution

The MIT-BIH arrhythmia dataset, widely utilized in arrhythmia classification research, comprises recordings from 47 individuals, each contributing a roughly 30 min arrhythmia recording. This dataset encapsulates a total of 109,500 cardiac beats, with approximately 30% classified as abnormal beats. It includes five types of cardiac beats: normal beats (N), atrial premature beats (A), ventricular premature beats (V), left bundle-branch block (L), and right bundle-branch block (R). Its validity has been established, making it a benchmark dataset in the study of cardiac arrhythmias.

In this experiment, all cyclic waveforms collected were referenced to the R-peak identified within the dataset. One hundred time points were captured before the R-peak, and two hundred time points were captured following the R-peak. Thus, a complete cyclic waveform was constructed through these three hundred time points.

In our study, we applied wavelet transform techniques to denoise signals in the MIT-BIH arrhythmia database, aiming to enhance the quality of the electrocardiogram (ECG) signals. We chose the fifth-order Daubechies wavelet as the mother wavelet function due to its excellent signal processing characteristics in biomedical signal analysis. By setting the decomposition level to nine, we obtained nine levels of detail coefficients (cD9 to cD1) and one level of approximation coefficient (cA9), allowing for a more refined analysis of the signal’s frequency characteristics and noise components. For determining the threshold in the denoising process, we adopted the VisuShrink threshold calculation formula, a method capable of adaptively adjusting the threshold size based on the characteristics of the data itself, effectively removing noise while preserving important signal features. This adaptive approach is suitable for processing signals with various noise levels, demonstrating good results in ECG signal denoising, where the average signal-to-noise ratio improved to 23.59031. We also compiled the SNR for each patient in Table 1, detailing the effectiveness of our denoising process across individual cases.

**Table 1:** SNR for patients in the MIT-BIH dataset.

Patient ID	SNR	Patient ID	SNR	Patient ID	SNR	Patient ID	SNR	Patient ID	SNR
100	17.4781	113	21.0052	200	23.2893	215	19.9525	232	20.0286
101	20.3131	114	23.1419	201	23.2442	217	25.8016	233	29.3282
103	21.6856	115	21.1164	202	24.2503	219	28.4727	234	25.3131
105	25.5353	116	24.9867	203	23.6702	220	18.7853		
106	21.8475	117	22.8157	205	20.0399	221	22.9026		
107	27.7311	119	26.3884	208	24.5882	222	17.6617		
108	22.5713	121	27.3271	210	22.7814	223	26.2516		
109	28.8873	122	24.3446	212	19.1808	228	23.6449		
111	22.9275	123	21.7020	213	29.4519	230	24.5426		
112	23.3215	124	29.5289	214	25.9638	231	20.5810		

Additionally, our threshold calculation formula is as follows:

$$\lambda = \sigma \sqrt {2lnN} ,$$

$$\sigma = \frac{MAD}{{0.6745}}$$

where $MAD$ represents the median of the absolute deviations from the median of the wavelet coefficients across all high-frequency subbands.

**Figure 1:**
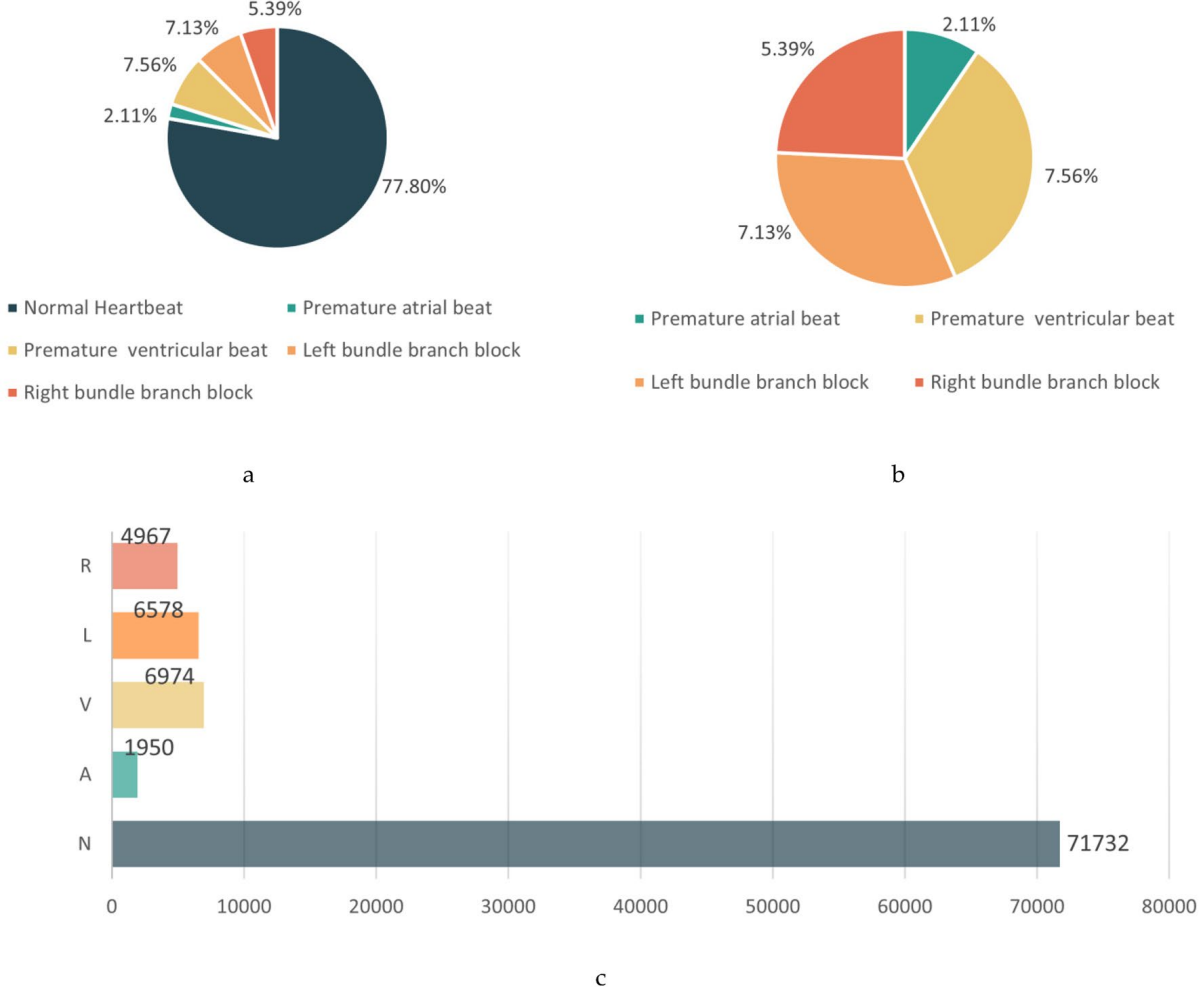
Plot of the original dataset analysis. (**a**) Original dataset distribution. (**b**) Percentage of scarcity data. (**c**) Original data bar graph distribution.

Figure 1 presents a detailed analysis of the MIT-BIH dataset. Following preprocessing, abnormal heart rates constituted 23% of the entire dataset. Notably, the premature atrial beat category represented a notably smaller portion, comprising only 2.006% of the total sample size.

**Figure 2:**
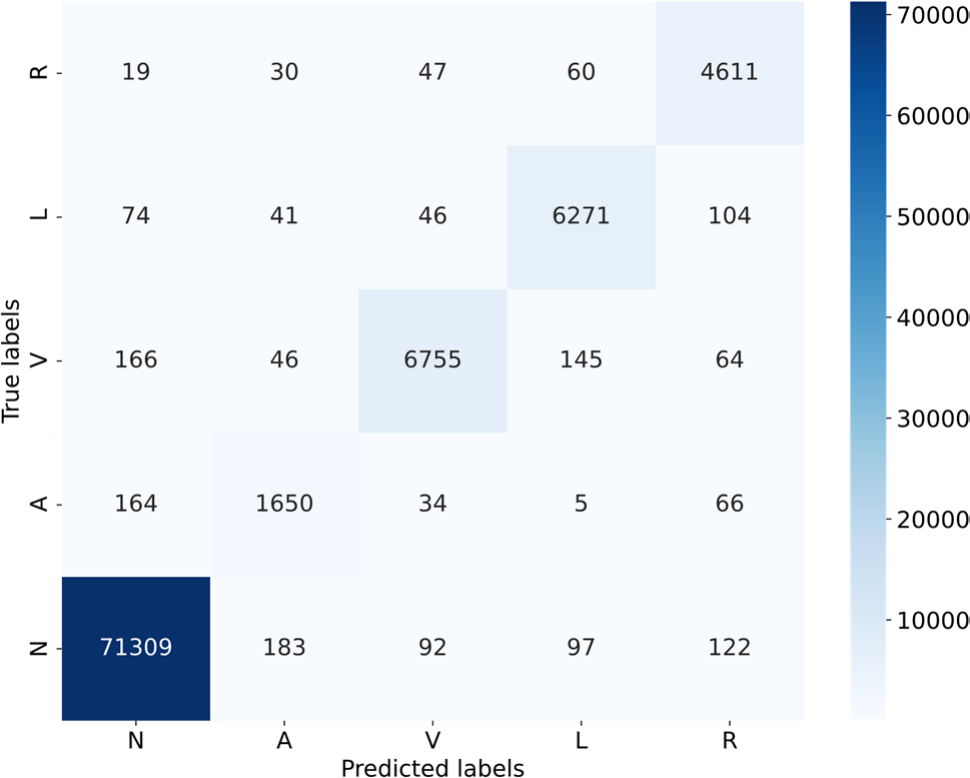
Classification model confusion matrix.

**Figure 3:**
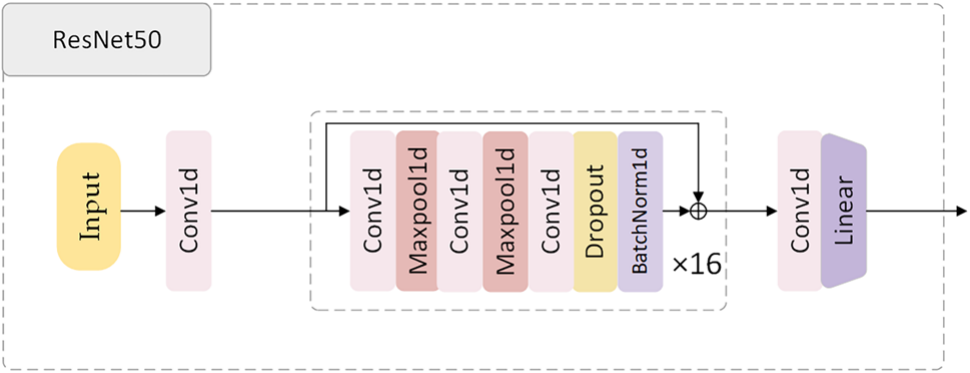
Structural diagram of the ResNet50 classification algorithm.

**Figure 4:**
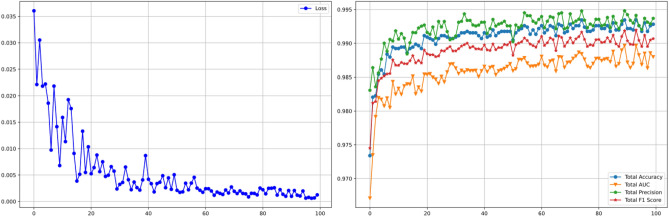
ResNet-50 classification metric curves.

We employed the ResNet50 algorithm for classifying heart rate data within the dataset. Given the characteristics of heart rate data, the unique residual units of ResNet allow gradients to propagate directly to earlier layers, effectively preventing the vanishing gradient problem often encountered when training deep networks. This architecture comprises 16 residual blocks in total, with each block containing three residual units and one additional residual unit attached to both the input and output of each block. This design ensures rapid training speeds while maintaining high training efficiency. The test results of the ResNet50 we designed are shown in Fig. 2 and Table 2. The structure diagram of ResNet50 is shown in Fig. 3. The model parameters and hyperparameters of the ResNet50 algorithm are detailed in Table 3, and the loss curves and metric curves during the training process are shown in Fig. 4. Results, indicate poor performance of scarce samples in the dataset during classification. Specifically, there is a significant variance in classification accuracy among four types of anomaly data, with the lowest at 85.982% and the highest at 96.727%. This notable variation underscores deficiencies in the classification outcomes, leading to decreased precision, recall, and F1 scores. Hence, particularly in cases of limited sample size, the presence of anomalous data in unbalanced datasets increases the risk of misdiagnosis or oversight (Supplementary Information).

**Table 2:** Former MIT-BIH classification results.

Serial number	Label	Precision	Recall	F1 score
1	Normal heartbeat	99.3120	99.4103	99.3611
2	Premature atrial beat	85.9823	84.6154	85.2934
3	Premature ventricular beat	94.1332	96.8598	95.4770
4	Left bundle branch block	95.9455	95.3329	95.6382
5	Right bundle branch block	96.7275	92.8327	94.7401
	Avg	94.4201	93.8102	94.1020

**Table 3:** ResNet50 model parameter settings.

Block	Layer	Kernel	Padding	Input size	Output size
Preprocess	PCA	/		256 × 300 × 1	256 × 16 × 1
	Conv1	3	1	256 × 16 × 1	256 × 16 × 64
	BatchNorm	/		256 × 16 × 64	256 × 16 × 64
	Relu	/		256 × 16 × 64	256 × 16 × 64
Layer1	ResBlock1	5	2	256 × 16 × 64	256 × 16 × 128
	ResBlock2 ~ 8	5	2	256 × 16 × 128	256 × 16 × 128
Layer2	ResBlock1	3	1	256 × 16 × 128	256 × 16 × 256
	ResBlock2 ~ 8	3	1	256 × 16 × 256	256 × 16 × 256
Classify	Conv2	3	1	256 × 16 × 256	256 × 16 × 256
	Fc	/		256 × 1024	256 × 5

Parameter	Value	Parameter	Value	Parameter	Value
Batch size	256	Learning rate	1e-4	epochs	100
Optimizer	Adam	Loss function	BCELoss	Conv per block	3
Train rate	0.8

In the medical domain, achieving high accuracy in recognizing abnormal heart rate data is of paramount importance, given that missed detections could gravely affect a patient’s life and health. Consequently, addressing the dataset imbalance and thereby enhancing the recognition accuracy of abnormal data represents the central issue and challenge of this study.

### Solution strategy of abnormal heart rate dataset based on CGAN

**Figure 5:**
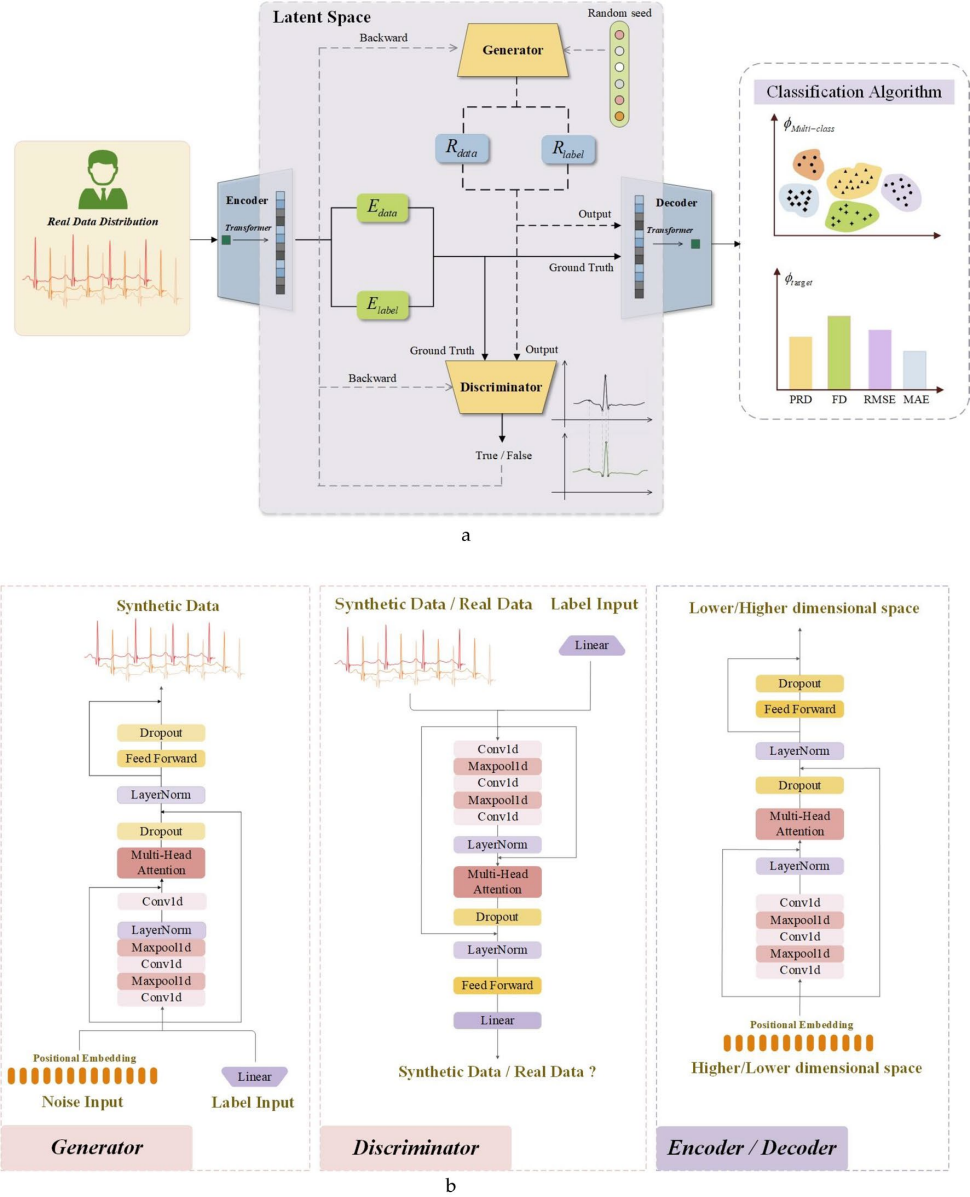
CECG-GAN-based strategy for addressing sparse anomaly in heart rate datasets. (**a**) General model diagram. (**b**) CECG-GAN specific model diagram.

Building upon our thorough analysis of the unbalanced dataset, we propose a solution centered around CECG-GAN for addressing the imbalance in heart rate data, as illustrated in Fig. 5. This approach specifically targets the issue of scarce anomalous data. The network’s design is inspired by the training methodology of TimeGAN^1^, which involves mapping high-dimensional data to a lower-dimensional space, thereby facilitating more effective model learning.

$H = \{ (H_{data}^{[n]} ,H_{label}^{[n]} )\}_{n = 1}^{N}$ , $H_{data} \in {\mathbb{R}}^{n \times l \times f}$ in Fig. 5a represents the original data in the high-dimensional space, where $l$ represents the length of the sample sequence and $f$ represents the number of features, and since the heart rate data is a one-dimensional time-series data, $f = 1$.$H_{label} \in {\mathbb{R}}^{n \times classes}$ denotes original data labels in high dimensional space, $E = \{ (E_{data}^{[n]} ,E_{label}^{[n]} )\}_{n = 1}^{N}$ denotes original data and labels in low dimensional space, and $R = \{ (R_{data}^{[n]} ,R_{label}^{[n]} )\}_{n = 1}^{N}$ denotes synthetic data and labels in low dimensional space.

As illustrated in Fig. 5b, the CECG-GAN framework encompasses four essential modules: the encoder, decoder, generator, and discriminator. The comprehensive execution process of the model’s algorithm is methodically detailed in Table 4. The primary objective of training this algorithm is to generate a synthetic dataset specifically designed to augment the sparse samples present in dataset $S = \{ (S_{data}^{(m)} ,S_{label}^{(m)} )\}_{m = 1}^{M}$*.*

**Table 4:** CECG-GAN model implementation flow.

Algorithm: CECG-GAN	
Input: ECG Training Set $H = \{ (H_{data}^{[n]} ,H_{label}^{[n]} )\}_{n = 1}^{N}$ , the maximum number of iterations T 1. ***repeat*** 2. *for* $n = 1 \cdots N$ ***do*** 3. Step1. Training Data $H_{data}^{[n]}$ to Develop Reversible Mapping Capability from High-Dimensional to Low-Dimensional Space 4. Step2. Training the Generator to Minimize Discrepancies Relative to the Encoder 5. Step3. Training generator and discriminator to improve generation effectiveness 6. Step4. Combining conditional constraints to generate scarce datasets $S = \{ (S_{data}^{(m)} ,S_{label}^{(m)} )\}_{m = 1}^{M}$ 7. $PRD \leftarrow \sqrt {\frac{{\sum {_{n = 1}^{N} (H_{data}^{[n]} - S_{data}^{[n]} )^{2} } }}{{\sum {_{n = 1}^{N} } (H_{data}^{[n]} )^{2} }}}$ 8. $FD \leftarrow \mathop {\min }\limits_{n = 1,...N} \{ \max (H_{data}^{[n]} ,S_{data}^{[n]} )\}$ 9. $RMSE \leftarrow \sqrt {\frac{1}{N}\sum {_{n = 1}^{N} (H_{data}^{[n]} - S_{data}^{[n]} )}^{2} }$ 10. $MAE \leftarrow \frac{1}{N}\sum {_{n = 1}^{N} |H_{data}^{[n]} - S_{data}^{[n]} |}$ 11. *t* = *t* + *1* 12. ***until t* = *T break*** Output: Synthetic dataset $S = \{ (S_{data}^{(m)} ,S_{label}^{(m)} )\}_{m = 1}^{M}$	

The encoder and decoder within the model are utilized to establish an invertible mapping between the high-dimensional $H_{data}$ and low-dimensional $E_{data}$ representations of the ECG signal $H \Leftrightarrow E$. This configuration enables the model to more effectively capture the signal’s characteristics $E_{data}$ in a low-dimensional space. Additionally, it facilitates the decoding and recovery of $E_{data}$ back to $H_{data}$, allowing for the accurate reconstruction of the original ECG signal.

The generator in the CECG-GAN model initiates by sampling from Gaussian noise and learns the signal characteristics $E_{data}$ of the original high-dimensional data $H_{data}$ within a low-dimensional space. Concurrently, the discriminator’s objective is to maximize its accuracy in identifying real data while minimizing its accuracy on the synthetic data generated by the generator. Through multiple iterations, the discriminator develops the capability to distinguish between real and synthetic data effectively. Simultaneously, the Generator refines its strategy, progressively producing results more closely resembling the actual data.

To address the slow training speed characteristic of existing models, all four modules in the CECG-GAN—encoder, decoder, generator, and discriminator—are built using transformer architectures. This design enables parallel data output, a significant departure from the sequential output typical of traditional recurrent neural networks. As a result, the model achieves substantially faster processing speeds.

Initially, the data undergoes positional encoding to integrate positional information, which is essential for context-aware processing. Subsequently, local and global features are extracted through multi-scale convolution, effectively capturing different aspects of the data. To optimize computational efficiency while preserving key data characteristics, maximum pooling is employed. Furthermore, the integration of a multi-attention mechanism enhances the model’s capacity for comprehending and representing the input data, concentrating on both local and global features. Additionally, the incorporation of residual connectivity within the model ensures that global features are retained while learning specific local features, thereby maintaining a balance between detailed and overarching data characteristics.

In our approach, both the generator and discriminator integrate data labels $H_{label}$ into the feature matrix, serving as conditional constraints for controlled generation of the target waveform. The retention of the dropout module not only prevents model overfitting but also facilitates increased diversity in the synthetic data $H_{data}$. The inclusion of $H_{label}$ significantly enhances the generator’s effectiveness and directs its generation process towards optimal data fitting. Through numerous iterations, the Encoder and decoder modules progressively learn the reversible mapping $E_{data}$ from high-dimensional to low-dimensional space. Meanwhile, the generator and discriminator gradually approach a Nash equilibrium state. Ultimately, the generator is capable of producing an ECG signal $S_{data}$ that closely resembles the original data $H_{data}$.

### The selection of synthetic ECG samples

**Figure 6:**
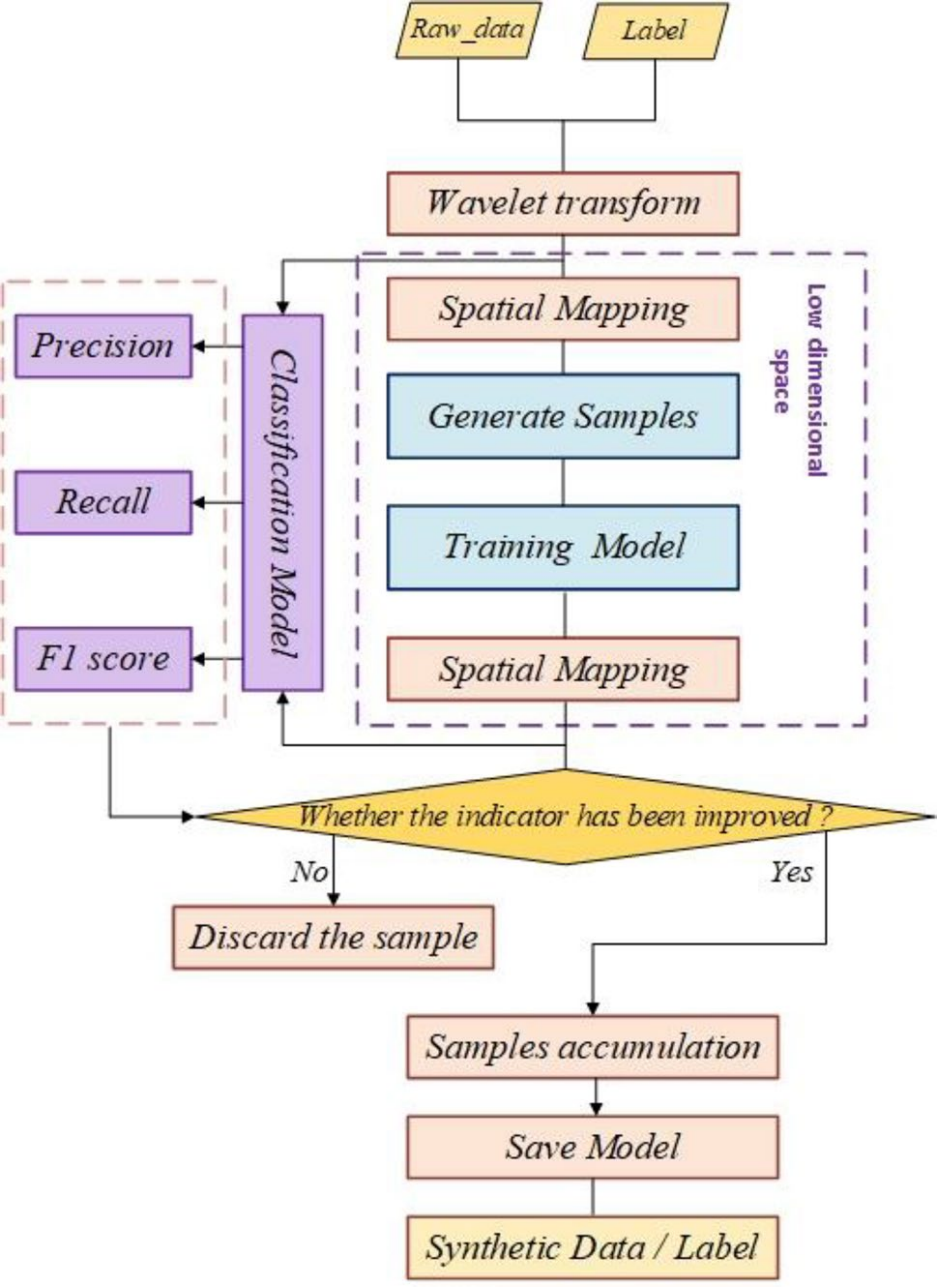
The screening process for synthetic samples.

The screening process of synthetic samples is shown in Fig. 6, the original dataset is filtered out the noise by wavelet transform, subsequently, the high dimensional data is mapped to the low dimensional space representation. After the model is trained, the synthetic data captured by the model is mapped to the high dimensional space and is given to the classification judgement model, if there is an improvement in the precision rate, recall rate, F1-score, etc. as compared to the original dataset, it is judged to be a valid sample and is saved.

### Ethics approval and consent to participate

This study utilizes two publicly available medical datasets: the MIT-BIH arrhythmia database and the China physiological signal challenge 2020 (CPSC 2020) dataset.

The MIT-BIH arrhythmia database was created through a collaboration between the Massachusetts Institute of Technology (MIT) and the Boston Beth Israel Hospital (BIH). It is recognized as the standard reference in the study of arrhythmias and electrocardiography (ECG). The database comprises ECG recordings from 47 individuals, thoroughly annotated and made publicly available for academic and research purposes. The creation and use of the MIT-BIH database complied with the ethical standards of the time, with all participants being fully informed and consenting to the use of their data for scientific research prior to their involvement.

The China physiological signal challenge 2020 (CPSC 2020) dataset is provided by the organizing committee of the China physiological signal challenge, aimed at advancing research in the field of arrhythmia detection. This database contains a substantial number of ECG records from various individuals, standardized for academic research purposes. The collection, organization, and dissemination of this database adhered to the relevant ethical guidelines and procedures, with all participants being informed and consenting to the use of their data for scientific purposes prior to data collection.

In our study, we strictly abide by the usage regulations set by the providers of these datasets, ensuring that the data is used solely for non-commercial scientific research. Moreover, we respect the confidentiality and anonymity of all data, ensuring that no personally identifiable information is disclosed in the course of our research.

## Results and discussion

### Experimental environment and parameter settings

The computer system is Windows 10.The software conditions include python3.7 and pytorch1.13 as the experimental framework, and the hardware conditions include Intel(R) Core (TM) i9-10920X CPU@3.50 GHz, equipped with 128 G of operating memory, NVIDIA Geforce RTX 3090 GPU.

In the training process, each network of the model adopts the transformer structure, using the multi-head attention mechanism in conjunction with the feed-forward network, fusing the residual connection network, so that the data combines the global information with the local information and output in parallel, in order to increase the depth of the model at the same time do not lose the data information, take the LayerNorm layer to normalise low-dimensional data, the specific model parameter table is shown in Table 5.

**Table 5:** List of model specific parameters.

Model	Layer	Kernel/head	Input size	Output size	Model	Layer	Kernel/head	Input size	Output size
Encoder	Conv1	1	128 × 100 × 1	128 × 100 × 64	Decoder	Conv1	1	128 × 100 × 512	128 × 100 × 512
	Maxpool1	1	128 × 100 × 64	128 × 100 × 64		Maxpool1	1	128 × 100 × 512	128 × 100 × 512
	Conv2	3	128 × 100 × 64	128 × 100 × 128		Conv2	3	128 × 100 × 512	128 × 100 × 512
	Maxpool2	3	128 × 100 × 128	128 × 100 × 128		Maxpool2	3	128 × 100 × 512	128 × 100 × 512
	Conv3	5	128 × 100 × 128	128 × 100 × 512		Conv3	5	128 × 100 × 512	128 × 100 × 512
	Norm1	–	128 × 100 × 512	128 × 100 × 512		Norm1	–	128 × 100 × 512	128 × 100 × 512
	Multi-Head Atten	8	128 × 100 × 512	128 × 100 × 512		Multi-Head Atten	8	128 × 100 × 512	128 × 100 × 512
	Norm2	–	128 × 100 × 512	128 × 100 × 512		Norm2	–	128 × 100 × 512	128 × 100 × 512
	Fc1	–	128 × 100 × 512	128 × 100 × 1024		Fc1	–	128 × 100 × 512	128 × 100 × 1024
	Norm3	–	128 × 100 × 1024	128 × 100 × 1024		Norm3	–	128 × 100 × 1024	128 × 100 × 1024
	Fc2	–	128 × 100 × 1024	128 × 100 × 512		Fc2	–	128 × 100 × 1024	128 × 100 × 1
Generator	Conv1	1	128 × 100 × 512	128 × 100 × 512	Discriminator	Conv1	1	128 × 100 × 1	128 × 100 × 64
	Maxpool1	1	128 × 100 × 512	128 × 100 × 512		Maxpool1	1	128 × 100 × 64	128 × 100 × 64
	Conv2	3	128 × 100 × 512	128 × 100 × 512		Conv2	3	128 × 100 × 64	128 × 100 × 128
	Maxpool2	3	128 × 100 × 512	128 × 100 × 512		Maxpool2	3	128 × 100 × 128	128 × 100 × 128
	Conv3	5	128 × 100 × 512	128 × 100 × 512		Conv3	5	128 × 100 × 128	128 × 100 × 512
	Norm1	–	128 × 100 × 512	128 × 100 × 512		Norm1	–	128 × 100 × 512	128 × 100 × 512
	Multi-Head Atten	8	128 × 100 × 512	128 × 100 × 512		Multi-Head Atten	8	128 × 100 × 512	128 × 100 × 512
	Norm2	–	128 × 100 × 512	128 × 100 × 512		Norm2	–	128 × 100 × 512	128 × 100 × 512
	Fc1	–	128 × 100 × 512	128 × 100 × 1024		Fc1	–	128 × 100 × 512	128 × 100 × 1024
	Norm3	–	128 × 100 × 1024	128 × 100 × 1024		Norm3	–	128 × 100 × 1024	128 × 100 × 1024
	Fc2	–	128 × 100 × 1024	128 × 100 × 512		Fc2	–	128 × 100 × 1024	128 × 5

### Comparison of experimental results with comparable models

In the training process, CECG-GAN adopts the strategy of alternately training the generator and the discriminator, in order to ensure the dynamic balance between the generator and the discriminator, in each iteration, the number of times the generator and the discriminator are trained in relation to each other is 2:1, and by splicing the inputs of the generator and the discriminator with the conditional vectors $H_{label}$, the model is capable of generating the corresponding types of waveform curves under the given conditional constraints. The Adam optimiser is also chosen to optimise the model parameters and after several rounds of iterations, the model will converge and reach a stable region. Theoretically, at this point the generator of the model will have the ability to generate a distribution similar to the real data and map it to a higher dimensional space using the decoder, and the discriminator will have difficulty in distinguishing between the real data and the synthetic data.

**Figure 7:**
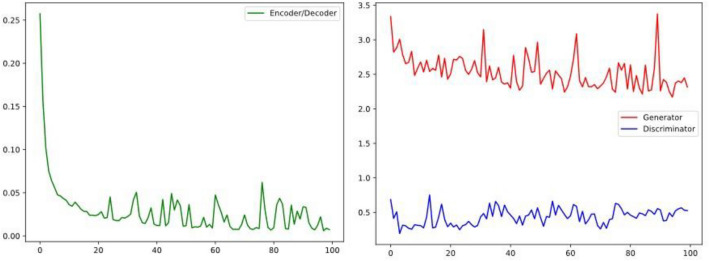
Loss function curve of generator and discriminator.

As shown in Fig. 7, the model gradually reaches stability after 100 epochs, at which point the network model structure achieves relatively excellent training results. In principle, at this time, the generator generated by the “fake” samples are no longer quickly identified, the samples have a similar distribution to the real samples, at this time to save the model network parameters, to facilitate the subsequent call at any time and generate the distribution of imbalance of the data, so as to effectively expand the dataset, so that the network model has the ability to ensure the authenticity of the samples and the expansion of the efficiency. Meanwhile, in order to verify the feasibility and validity of our proposed model, the experiment adopts the K-fold validation method, where multiple rounds of testing and validation of the model are carried out using different data subsets, where the model is trained on each of the K data subsets and a comprehensive assessment of the model performance is obtained. As shown in Table 6, the model performs best in the nine-fold validation, achieving PRD of 21.138, FD of 0.695, and RMSE of 0.071, which are results that indicate that our model is able to show stable convergence during iterations, further confirming its validity.

**Table 6:** Parameters of the K-fold validation model (for patient 100).

K-fold	PRD	FD	RMSE
K = 5	27.598	0.838	0.096
K = 6	25.347	0.811	0.088
K = 7	25.618	0.904	0.091
K = 8	24.309	0.817	0.102
K = 9	21.138	0.695	0.071

**Figure 8:**
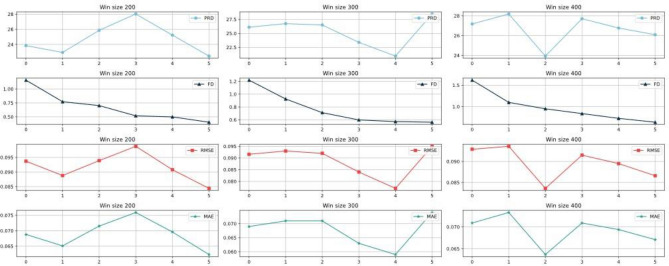
Metrics results for different length windows.

Since the MIT-BIH dataset has a sampling rate of 360 Hz and a typical cardiac cycle typically lasts 0.6–1 s, setting a window size of 200–400 captures a complete heart rate waveform. As shown in Fig. 8, we explored the effect of different window sizes on the training results. The experimental design consists of two scenarios: either the window size is fixed with different sampling intervals, or the window size is different but the sampling intervals are the same. The results show that increasing the sampling interval leads to a decrease in performance metrics such as PRD, FD, RMSE and MAE when the window size is kept consistent. However, too high a sampling interval may miss key features of heart rate. Therefore, we believe that a sample interval setting of 3 or 4 provides the best results.

**Figure 9:**
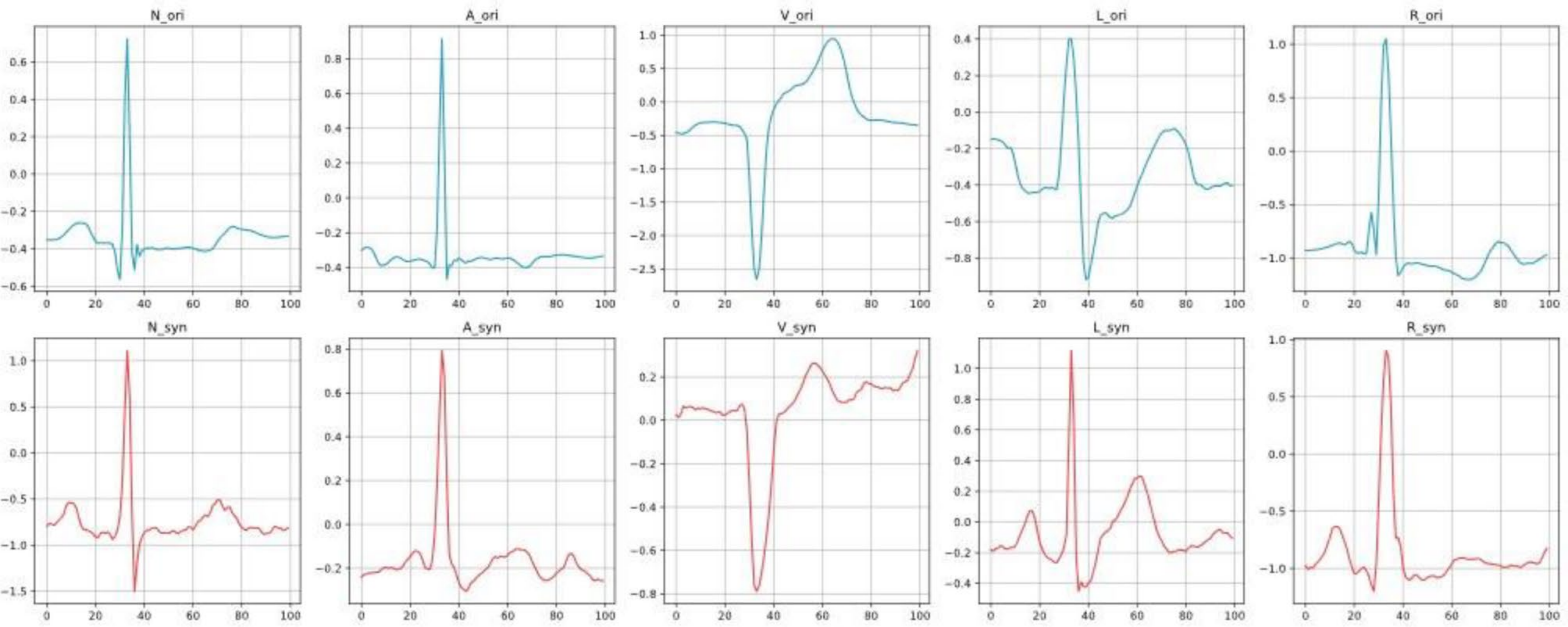
Visualisation of the results of the MIT-BIH “faked” ECG signal samples (in patients 100, 105, 109, 124 combined training).

At the same time, we visualised the “faked” samples, as shown in Fig. 9, taking into account the effect of factors such as resting heart rate between patients. In the experiment, it was found that too many patients participated in the training will lead to too poor results, and the single patient dataset is not satisfied to contain all the scarce samples, so this result was jointly trained by five patients to generate examples containing all kinds of forged samples, and at the same time, they are compared with the real data, and it can be intuitively seen that synthetic data and real data visually present a similar signal distribution, and therefore it verifies the validity of our proposed model in the perspective of heart rate generation.

**Figure 10:**
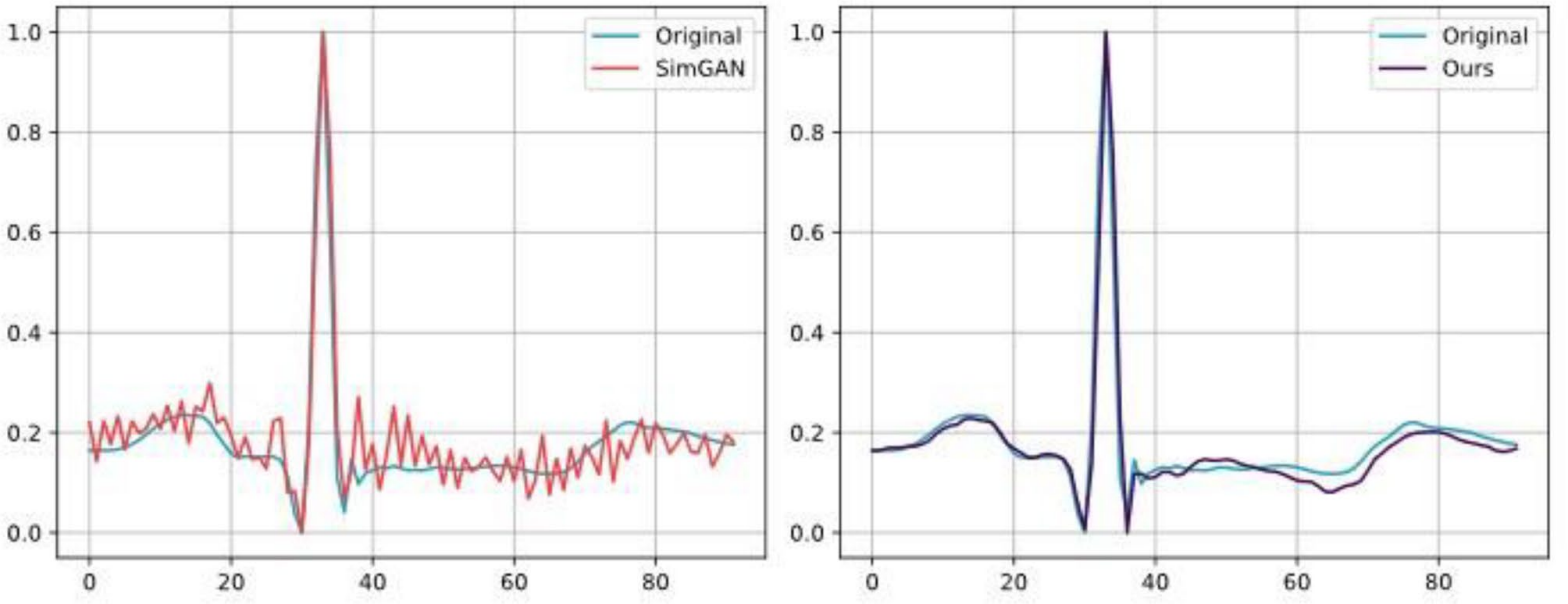
Comparison of the effect of CECG-GAN and SimGAN in synthesising lead ECG signals (compared with patient 100).

At the same time, we compared the model effect with the same kind of model, as shown in Fig. 10. We used a combination of multi-scale convolutional kernels to remove a lot of jitter from the generation effect, and compared to the SimGAN model, which synthesises signals with considerable noise, our synthesised signals have very little noise and higher synthesis quality.

As shown in Table 7, we performed single-patient testing on all patients in the MIT-BIH dataset, with most of them performing well, while at the same time, due to the superimposed effects of physiological differences between patients and the type of disease, there were also many patients who did not perform well, with an average PRD of 55.048, FD of 1.139, RMSE of 0.232, and MAE of 0.166.

**Table 7:** MIT-BIH dataset single patient experiments.

Patient id	PRD	FD	RMSE	MAE	Patient id	PRD	FD	RMSE	MAE
100	20.938	0.572	0.077	0.059	202	49.455	1.219	0.165	0.125
101	42.739	0.691	0.16	0.124	203	119.831	1.362	0.435	0.329
103	30.728	1.338	0.116	0.142	205	31.498	1.031	0.11	0.109
105	64.104	0.949	0.182	0.123	208	100.531	1.419	0.45	0.372
106	56.183	1.528	0.211	0.203	210	62.536	0.906	0.162	0.13
107	48.712	1.061	0.181	0.141	212	81.061	0.855	0.19	0.171
108	87.942	0.836	0.237	0.178	213	73.774	1.778	0.345	0.226
109	31.194	0.594	0.167	0.116	214	54.817	0.89	0.217	0.147
111	59.451	0.737	0.148	0.101	215	72.422	0.907	0.205	0.123
112	14.183	0.542	0.115	0.092	217	66.492	1.509	0.541	0.401
113	62.017	1.357	0.262	0.124	219	45.519	2.095	0.362	0.212
114	50.356	1.271	0.192	0.125	220	18.887	1.156	0.115	0.136
115	52.908	1.341	0.295	0.151	221	59.768	0.825	0.191	0.134
116	37.726	2.516	0.431	0.274	222	71.066	0.512	0.133	0.103
117	22.429	1.009	0.185	0.128	223	33.821	1.647	0.181	0.161
119	30.779	1.661	0.32	0.176	228	124.582	0.76	0.442	0.27
121	36.419	0.741	0.298	0.225	230	58.559	1.614	0.211	0.188
122	13.799	0.853	0.126	0.087	231	32.088	0.665	0.104	0.094
123	13.427	0.877	0.108	0.064	232	61.878	0.625	0.131	0.165
124	31.361	1.871	0.38	0.183	233	129.516	1.617	0.493	0.333
200	85.26	1.335	0.347	0.174	234	56.235	1.163	0.185	0.155
201	70.093	0.744	0.102	0.098	Avg	**55.048**	**1.139**	**0.232**	**0.166**

Significant values are given in bold.

Further, we compare and analyse the model with the previous approach with many benchmark models, as shown in Table 8, our model is far better than other models in PRD, RMSE, and MAE metrics, which again verifies the feasibility of our proposed model and demonstrates the degree of superior performance of our model.

**Table 8:** Comparison of metric validation of different heart rate generation models.

Model	BILSTM-CNN GAN	RNN-AE GAN	LSTM-AE GAN	RNN-VAE GAN	LSTM-VAE GAN
PRD	66.408	121.877	148.650	146.566	145.978
FD	**0.756**	0.969	0.996	0.982	0.975
RMSE	0.276	0.506	0.618	0.609	0.607
MAE	0.501	0.795	0.771	0.794	0.714

Model	BiLSTM-GRU	BiLSTM-LSTM	BiLSTM-MLP	BiLSTM-CNN GAN	BiGridLSTM-CNN
PRD	74.047	84.795	147.732	57.168	66.211
FD	0.853	0.901	0.989	0.817	0.790
RMSE	0.308	0.352	0.614	0.231	0.251
MAE	0.597	0.668	0.751	0.500	0.366

Model	TimeGAN	TTS-GAN	Ours		
PRD	66.496	61.524	**55.048**		
FD	1.370	0.991	1.139		
RMSE	0.241	0.240	**0.232**		
MAE	0.279	0.268	**0.166**		

Significant values are given in bold.

### Comparative experiment of MIT-BIH dataset expansion effect analysis

In this study, to assess the performance improvements of imbalanced datasets before and after data augmentation, we have chosen the F1-Score as our primary evaluation metric. The F1-Score, being the harmonic mean of precision and recall, serves as a comprehensive indicator that can balance the model’s ability to identify and cover minority class samples. This is particularly applicable to the context of imbalanced datasets, as using either precision or recall alone may lead to biased evaluation results. For instance, a high precision rate might overlook the model’s capability to recognize minority classes, whereas a high recall rate could ignore the false positives generated by the model. Therefore, by balancing these two metrics, the F1-Score provides us with a more thorough and fair means to evaluate the effect of data augmentation on enhancing the performance of processing imbalanced datasets. In the comparative analysis before and after data augmentation, utilizing the F1-Score allows us to more precisely identify and validate the contribution of data augmentation techniques to improving the overall performance of the model, especially in terms of enhancing the model’s ability to recognize samples from minority classes.

$$F1 = 2 \times \frac{{\Pr ecision \times {\text{Re}} call}}{{\Pr ecision + {\text{Re}} call}}$$

**Figure 11:**
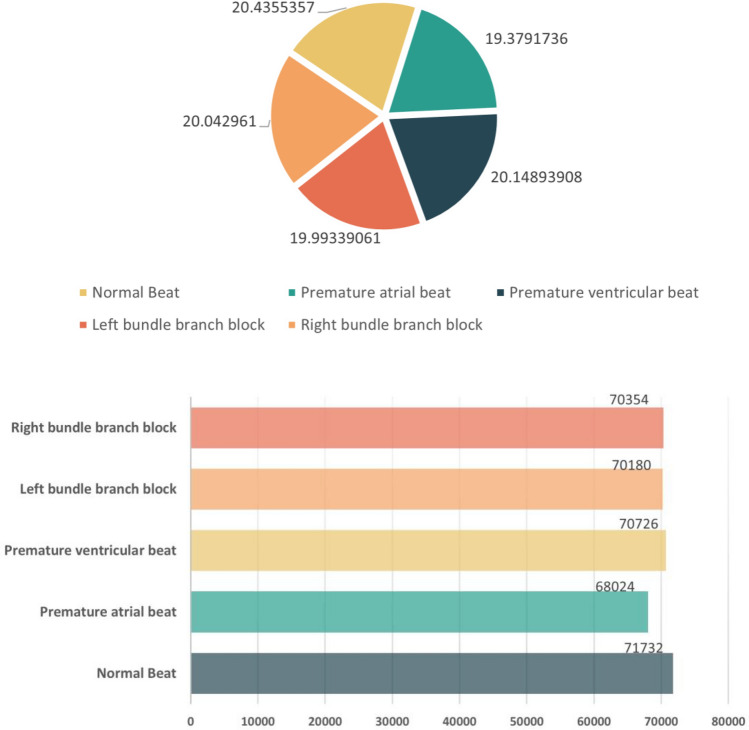
Scale analysis plot of the expanded dataset.

The MIT-BIH dataset contains four types of abnormal heart rates, namely, premature atrial beat, premature ventricular beat, left bundle branch block, and right bundle branch block, and after utilising our proposed model, the effect of the MIT-BIH dataset expansion is shown in Table 9 and Fig. 11. It is intuitively clear from the enlargement rates in the table that the more severe the unbalanced distribution in the original dataset, the higher the enlargement rate, which side-steps the necessity of our proposed modelling strategy.

**Table 9:** Effect of MIT-BIH dataset expansion.

Label	Before	After	Augmentation ratio (%)
Premature atrial beat	1950	68024	3488.41
Premature ventricular beat	6974	70726	1014.13
Left bundle branch block	6578	70180	1066.88
Right bundle branch block	4967	70354	1416.42

**Figure 12:**
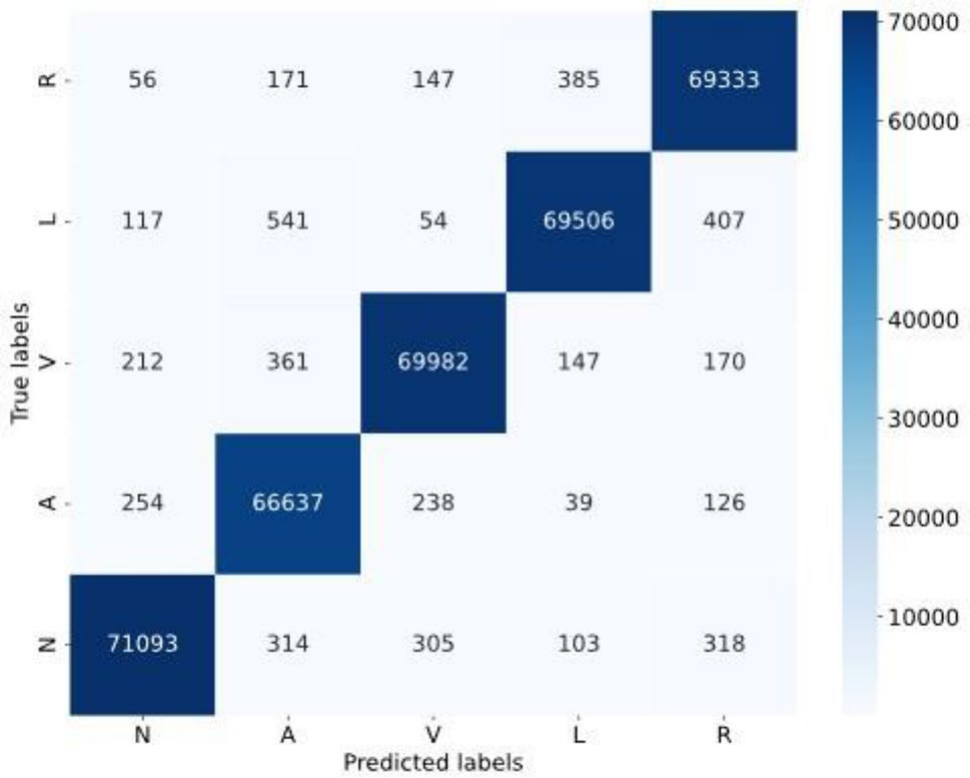
Confusion matrix analysis for the classification of the expanded dataset.

The revised paragraph effectively communicates the key findings as presented in Table 10 and Fig. 12. It highlights the substantial improvement in judgment accuracy and other metrics for scarce data within the expanded dataset, emphasizing that all category metrics exceed 98%. The paragraph also transparently addresses the trade-off involved in this enhancement, specifically the slight decrease in metrics for normal heart rate judgment. The conclusion that this minor reduction is considered acceptable against the backdrop of significant improvements in other areas is well articulated, maintaining a balanced perspective on the outcomes of the dataset expansion.

**Table 10:** Comparison of multi-classification results before and after the use of CECG-GAN heart rate generation model.

Serial number	Label	Precision	Ours-precision	Recall	Ours-recall
1	Normal Beat	**99.3120**	99.1092	**99.4103**	98.5582
2	Premature atrial beat	85.9823	**97.9610**	84.6154	**99.0237**
3	Premature ventricular beat	94.1332	**98.9481**	96.8598	**98.7442**
4	Left bundle branch block	95.9455	**99.0396**	95.3329	**98.4156**
5	Right bundle branch block	96.7275	**98.5488**	92.8327	**98.9171**
6	Avg	94.4201	**98.7213**	93.8102	**98.7318**

Serial Number	Label	F1 score	Ours-F1 score	Ori count	Expanded count
1	Normal Beat	**99.3611**	98.8329	71,732	71,732
2	Premature atrial beat	85.2934	**98.4965**	1950	68,024
3	Premature ventricular beat	95.4770	**98.8460**	6974	70,726
4	Left bundle branch block	95.6382	**98.7266**	6578	70,180
5	Right bundle branch block	94.7401	**98.7326**	4967	70,354
6	Avg	94.1020	**98.7255**		

Significant values are given in bold.

**Figure 13:**
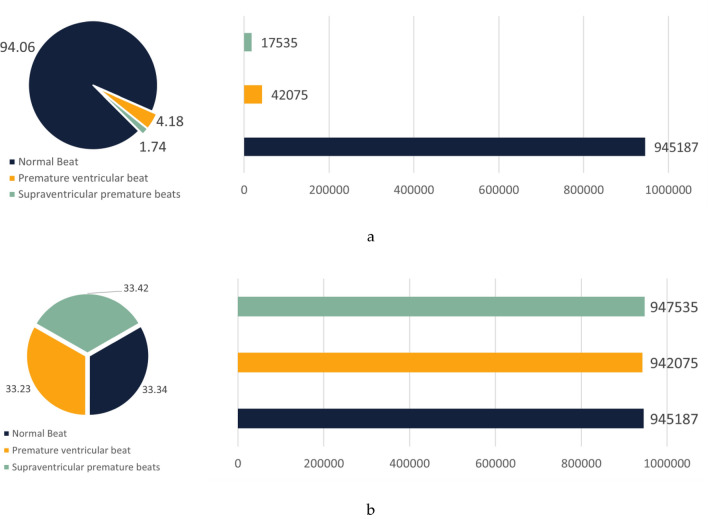
Comparison of CSPC2020 dataset analysis and expansion effect. (**a**) Distribution of the original CSPC2020 dataset; (**b**) effect of expanding the dataset using the CECG-GAN model.

To further validate the generalizability of the CECG-GAN model, additional experiments were conducted using the CSPC2020 dataset. This dataset encompasses three heart rate types: normal beat (N), premature ventricular beat (V), and supraventricular premature beats (S). Figure 13 highlights the dataset’s initial unbalanced distribution and the subsequent balancing effect achieved through the application of the CECG-GAN model. Comprising data from ten patients, each with approximately 24 h of recorded data, the CSPC2020 dataset is also recognized as a significant resource for cardiac arrhythmia research.

**Figure 14:**
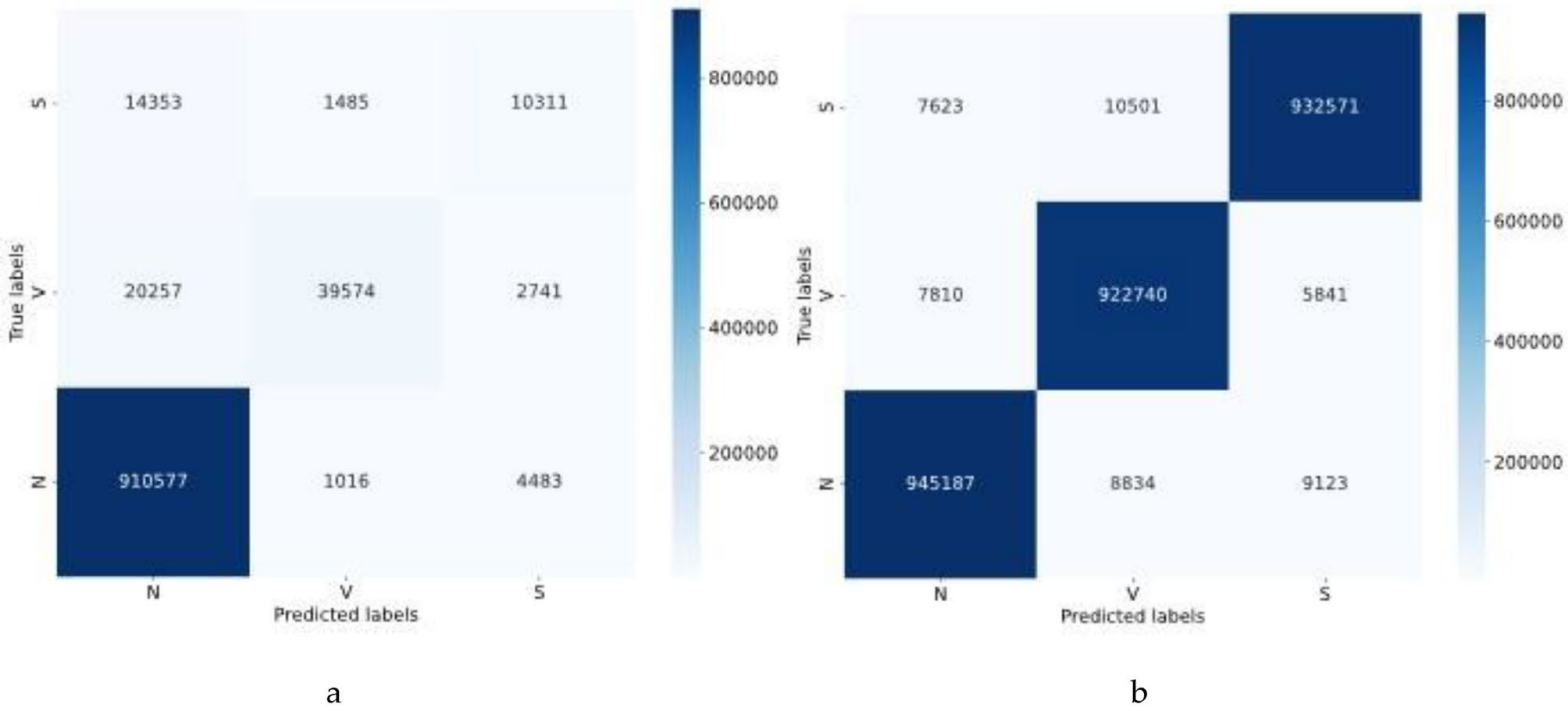
Comparison of confusion matrix before and after CSPC2020 expanded dataset, (**a**) confusion matrix before classification, (**b**) confusion matrix after dataset expansion.

The assessment of our model’s classification capabilities, both before and after its expansion, was conducted using a convolutional neural network. Table 11 details this comparative analysis, examining the change in metrics resulting from the expansion. Furthermore, an individual case study on the CSPC2020 dataset was carried out, with Fig. 14 showcasing the confusion matrices corresponding to the pre- and post-expansion stages. This approach further confirms the CECG-GAN model’s robustness and its wide applicability.

**Table 11:** Comparison of multiclassification results before and after expansion of the CSPC2020 dataset with accompanying single-patient experiments.

Serial number	Label	Precision	Ours	Recall	Ours	F1 score	Ours	Ori count	Expanded count
1	Normal beat	**0.9939**	0.9810	0.9633	**0.9836**	0.9784	**0.9823**	945,187	945,187
2	Premature ventricular beat	0.6324	**0.9854**	0.9405	**0.9794**	0.7563	**0.9824**	42,075	942,075
3	Supraventricular premature beats	0.3943	**0.9809**	0.5880	**0.9842**	0.4720	**0.9825**	17,535	947,535

ID	PRD	FD	RMSE	MAE	ID	PRD	FD	RMSE	MAE
1	55.407	0.841	0.09	0.061	6	48.712	0.709	0.155	0.113
2	32.114	0.54	0.074	0.058	7	98.335	1.054	0.224	0.099
3	31.877	0.498	0.116	0.042	8	27.994	0.688	0.149	0.088
4	81.203	0.904	0.165	0.117	9	74.109	0.507	0.114	0.067
5	48.227	0.661	0.167	0.085	10	14.183	0.305	0.055	0.049

Significant values are given in bold.

**Figure 15:**
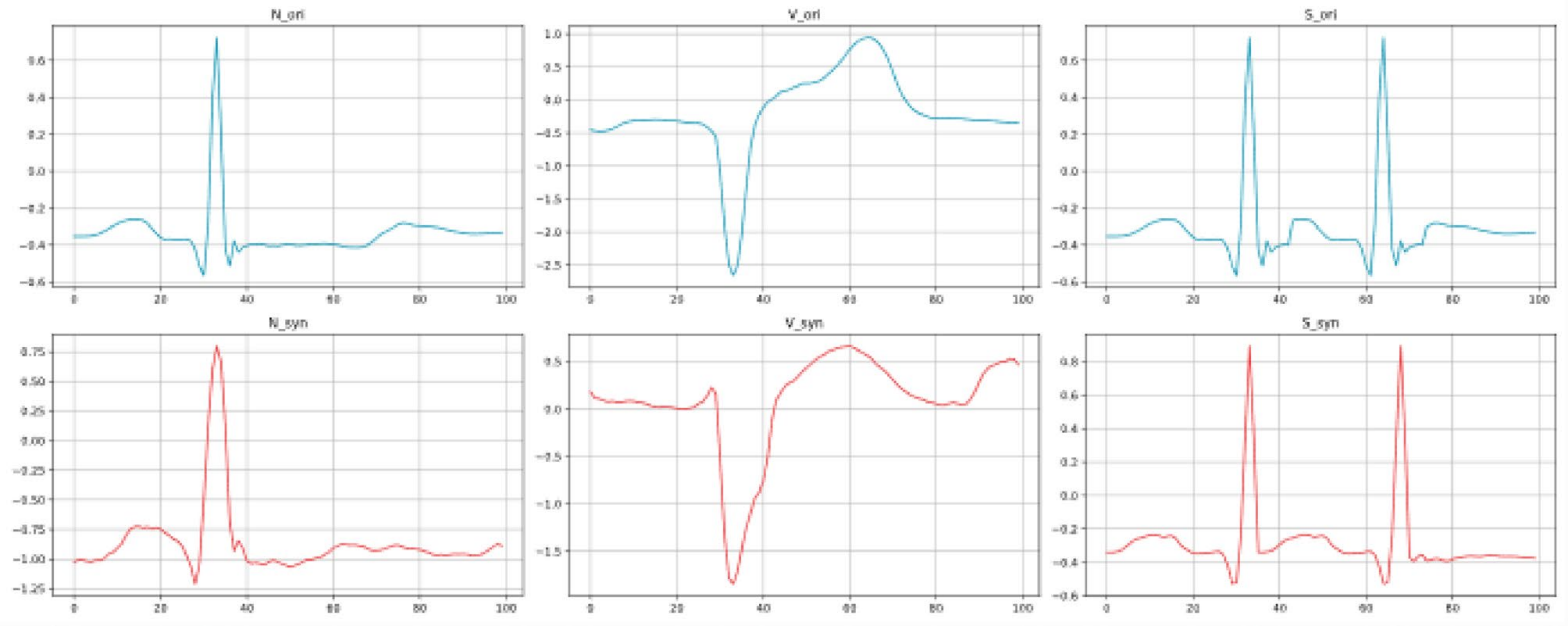
Visualization of ‘synthetic’ ECG signal samples with patient 04 as an example.

Simultaneously, we visualized the waveforms generated by the CECG-GAN model, which was trained on the CSPC2020 dataset, as depicted in Fig. 15. This figure presents the training outcomes for three distinct types of waveforms included in the dataset. The distributions produced by the model closely resemble those of the original dataset, thereby further confirming the generalizability and effectiveness of the CECG-GAN model.

In this study, two datasets were analyzed to identify critical issues. Firstly, the model’s primary objective is to enhance classification algorithm indices, necessitating the resolution of dataset imbalance. Current dataset expansion methods, predominantly using generative adversarial networks, offer the benefit of diversifying datasets by integrating noise with the model, while also safeguarding patient privacy. However, these models primarily focus on waveform generation, and the datasets employed (such as MIT-BIH and CSPC2020) typically exhibit significant category imbalances (with minimum category proportions at 2% and 1.74%, respectively). This leads to an imbalance in the samples synthesized by the model. The “synthetic fake samples” produced are overwhelmingly representative of normal heart rates, further aggravating the dataset’s imbalance issue. Additionally, existing models are largely based on recurrent neural networks, which are not optimal for long sequence tasks, resulting in prolonged training durations and high noise levels in the “synthetic fake samples”.

To address these issues, our proposed CECG-GAN model achieves parallel output based on the transformer architecture, which outputs all time steps at once with a short elapsed time, and also maps the data to the latent space learning through the invertible mapping technique, which effectively reduces the model noise and solves the problem of waveform jitter. In addition, we use conditional constraints to make the model controllably generate the specified type of heart rate, which successfully accomplishes the purpose of improving the index of the classification algorithm.

During our analysis, we encountered a significant challenge due to individual differences in baseline heart rates and disease conditions. A large training set hinders the model’s convergence, whereas a smaller set may not adequately represent all fundamental disease cases in the dataset. To mitigate this, we opted for a strategy where scarce heart rate data is generated by combining records from three to four individuals. This approach ensures the inclusion of all essential disease cases in the training set, thus enabling the model to accurately reflect the heart rates present in the dataset. However, it’s crucial to note that both datasets used in our study include only five main diseases. Consequently, the model’s performance in handling more complex disease scenarios has not been determined, presenting a potential limitation in its broader applicability.

## Conclusions

In conclusion, our study addresses the challenges of highly unbalanced datasets and prolonged computation times in heart disease research. We introduce a generative adversarial network algorithm that integrates low-dimensional space representation with a Transformer architecture. This innovation enables parallel output during model training, significantly reducing runtime. The model effectively synthesizes ECG data that closely mirrors the distribution of original ECG data. Furthermore, the implementation of conditional constraints allows for the generation of specific waveforms as required. In metric evaluations, our model surpasses existing algorithms in performance, particularly in mitigating the issue of jittery heart rate waveforms. The overall experimental results affirm the CECG-GAN’s viability and effectiveness in expanding heart rate datasets.

### Supplementary Information

Supplementary Information.

## Data Availability

The MIT-BIH and CSPC2020 datasets mentioned in this paper are both public datasets. They can be downloaded from the following addresses: https://www.physionet.org/content/mitdb/1.0.0/ and http://2020.icbeb.org/CSPC2020.
